# Could thyrotropin serum level characterize major depressive disorder phenotype? A systematic review and meta-analysis

**DOI:** 10.1007/s40618-025-02687-1

**Published:** 2025-08-19

**Authors:** Elisa Gatta, Virginia Maltese, Massimiliano Ugoccioni, Irene Silvestrini, Sara Corvaglia, Simone Vetrugno, Anna Ceraso, Antonio Vita, Mario Rotondi, Carlo Cappelli

**Affiliations:** 1 Department of Clinical and Experimental Sciences, SSD Endocrinologia, University of Brescia, ASST Spedali Civili, Brescia, Italy; 2 Centro per la Diagnosi e Cura delle Neoplasie Endocrine e delle Malattie della Tiroide, University of Brescia, Brescia, Italy; 3 Department of Internal Medicine and Therapeutics, University of Pavia, Pavia, Italy; 4 Department of Clinical and Experimental Sciences, Department of Mental Health and Addiction Services, University of Brescia, ASST Spedali Civili, Brescia, Italy; 5 Istituti Clinici Scientifici Maugeri IRCCS, Unit of Internal Medicine and Endocrinology, Laboratory for Endocrine Disruptors, Pavia, Italy; 6 Department of Clinical and Experimental Sciences, SSD Endocrinologia, University of Brescia, ASST Spedali Civili Brescia, Piazzale Spedali Civili n°1, Brescia, 25121 Italy

**Keywords:** Thyroid, Thyroid hormones, Major depressive disorder, Depression

## Abstract

**Purpose:**

Aim of this systematic review is to evaluate if and how thyrotropin (TSH) serum level levels may influence major depressive disorders (MDD) in drug naïve patients, and if it could characterize MDD phenotype.

**Methods:**

A PubMed/MEDLINE, Scopus and Web of Science databases was researched up to January 2025. The studies eligible addressed the questions define based on the PICO framework: (1) Are TSH levels different between first episode drug-naïve (FEDN) patients diagnosed with MDD and healthy subjects? (2) What are the TSH levels in FEDN patients diagnosed with MDD who attempt suicide compared to who do not? This review followed PRISMA guidelines. The quality assessment and the risk of bias were analyzed using QUADAS-2.

**Results:**

We included 45 studies in the qualitative synthesis, and 18 in the quantitative one, encompassing a total of 34,448 participants. Our systematic review showed conflicting data about TSH levels in FEDN MDD patients compared to healthy subjects. However, the meta-analysis showed in 6,224 patients that higher TSH levels are related to an increased risk of suicide attempt (Standardized Mean Difference = 1.848 mIU/L, C.I. 95%:1.506 to 2.190) with moderate-high heterogeneity across studies (I^2^ = 67%, *p* = .009).

**Conclusion:**

We showed conflicting data about TSH levels in FEDN MDD patients compared to healthy subjects. On the contrary, the meta-analysis evidenced significant higher TSH levels among MDD patients with suicide attempt than those without it. The clinical implications of this finding have yet to be established.

## Introduction

Thyroid stimulating hormone (TSH) is a critical biomarker for thyroid function, and its relationship with mood disorders, particularly Major Depressive Disorder (MDD), has garnered increasing attention in recent years [1, 2].The thyroid gland plays a vital role in regulating the body’s metabolic processes, and when its function is impaired, it can lead to a range of psychiatric symptoms [3]. Among these, depression is one of the most commonly observed manifestations in individuals with thyroid dysfunction [4].

MDD is a common yet complex mental health condition that affects millions of individuals representing the eleventh greatest cause of disability and mortality worldwide [5, 6]. In 2019, 7.8% (19.4 million) of adults in the US experienced at least 1 major depressive episode; 5.3% (13.1 million) experienced a major depressive episode with severe impairment [7, 8]. MDD is characterized by persistent feelings of sadness, hopelessness, and a lack of interest or pleasure in activities once enjoyed, severely impacting a person’s ability to function in daily life. Beyond emotional and psychological distress, depression often leads to other cognitive behavioral or neurovegetative symptoms [5, 9]. Depression can be a chronic condition characterized by periods of remission and recurrence, often beginning in adolescence or early adulthood ^7^. The MDD phenotype varies widely, with some individuals exhibiting a severe course marked by recurrent episodes and high comorbidity with anxiety disorders. A particularly concerning subset includes those with a history of suicide attempts, which is associated with greater symptom severity, impulsivity, and treatment resistance [10].

The exact causes of MDD are not fully understood, though several key factors contribute to its development. Genetic predisposition plays a substantial role, with individuals having a higher risk of depression if they have a family history of the disorder [11]. Neurobiological factors, particularly imbalances in neurotransmitters like serotonin, norepinephrine, and dopamine, are also central to the pathophysiology of depression. These chemical messengers influence mood regulation, and disruptions in their signaling pathways can lead to depressive symptoms [12].

Animal studies well established a close interplay between thyroid hormones and serotonin uptake as well as GABAergic activity [13, 14]. By the fact, hypothyroidism, characterized by low thyroid hormone levels, is often linked with symptoms of depression [4, 15], possibly due to impaired serotonin function [16]. On the other hand, GABA, the primary inhibitory neurotransmitter in the central nervous system, also interacts with thyroid hormones, modulating its activity by regulating GABA receptors and enzymes involved in its synthesis and breakdown [17]. Given the high prevalence of thyroid disfunction, characterized by TSH impairment, worldwide [18–23], it is quite common for individuals to be affected by hyper- or hypothyroidism and MDD simultaneously. For this reason, we performed a systematic review of the existing literature in order to evaluate if and how TSH levels may influence MDD in drug naïve patients, and furthermore a meta-analysis aimed to investigate if thyrotropin serum level could characterize MDD phenotype.

## Materials and methods

### Search strategy and inclusion criteria

A wide literature search of the PubMed/MEDLINE, Scopus and Web of Science databases was made.

The review questions were define based on the “Population, Intervention, Comparator, Outcome” framework (PICO):

1. Are TSH levels (outcome) different between first episode drug-naïve (FEDN) patients diagnosed with MDD (patients/population) and healthy subjects (comparator)?
2. What are the TSH levels (intervention) in FEDN patients diagnosed with MDD (patients/population) who attempt suicide (outcome) compared to who do not (comparator)?

The algorithm used for the research was the following: (“thyroid”) AND [(“depression”) OR (“major depressive disorder”)] AND (“suicide”).

The search was updated until January 31, 2025. Only articles in English were considered, and preclinical studies, conference proceedings, reviews, or editorials were excluded. To expand our search, the references of the retrieved articles were also screened for additional papers.

### Eligibility criteria

The eligibility criteria were chosen taking into account the review question. Clinical studies reporting TSH levels in patients diagnosed with MDD were deemed eligible for inclusion in this systematic review. Exclusion criteria for the systematic review (qualitative analysis) were reviews, letters, comments, editorials on the topic of interest, case reports, or small case series (fewer than 5 enrolled patients) on the analyzed topic (as these articles are characterized by poor-quality evidence and are typically affected by publication bias), as well as original articles dealing with different fields of interest.

No studies including patients with known thyroid diseases were included in the quantitative analysis. In addition to the systematic review of human studies, an animal study search was conducted to provide a mechanistic understanding of the findings observed in humans. This approach aimed to explore the underlying biological processes and potential pathways that may explain the thyroid-psychiatric interactions observed in human studies. Relevant animal models were identified and analyzed to complement the human data, offering insights into the pathophysiological mechanisms that may contribute to these associations.

### Study selection

E.G. and V.M. independently read the titles and abstracts of the records generated by the search algorithm. They then determined which studies were eligible based on predefined criteria. Disagreements were resolved with a third reviewer (C.C.).

### Quality assessment

The quality assessment of these studies, including the risk of bias and applicability concerns, was carried out using Quality Assessment of Diagnostic Accuracy Studies version 2 (QUADAS-2) evaluation [24].

### Data extraction

The reviewers collected data from all of the included studies, taking advantage of full-text, tables, and supplemental material concerning general study information (authors, publication year, country, study design, funding sources), patients’ characteristics (sample size, age, clinical setting, diagnosis, therapies), and TSH levels. The main findings of the articles included in this review are reported in the Results section.

### Statistical analysis

The data from the included studies were utilized, considering each study’s relative importance, employing a random-effect statistical model, due to the high heterogeneity in the analyzed studies. Furthermore, the study included the provision of 95% confidence interval values, which were subsequently visually represented through forest plots. The I-square (I^2^) index, also known as the inconsistency index, was employed to assess the level of statistical heterogeneity within the papers included in the analysis. Statistical heterogeneity was considered significant if the I-square index exceeded 50%. The software OpenMeta [Analyst]^®^ (version 3.13), supported by the Agency for Healthcare Research and Quality (AHRQ) in Rockville, MD, USA, was utilized to calculate the pooled values of mean differences.

## Results

### Literature search

**Fig. 1:**
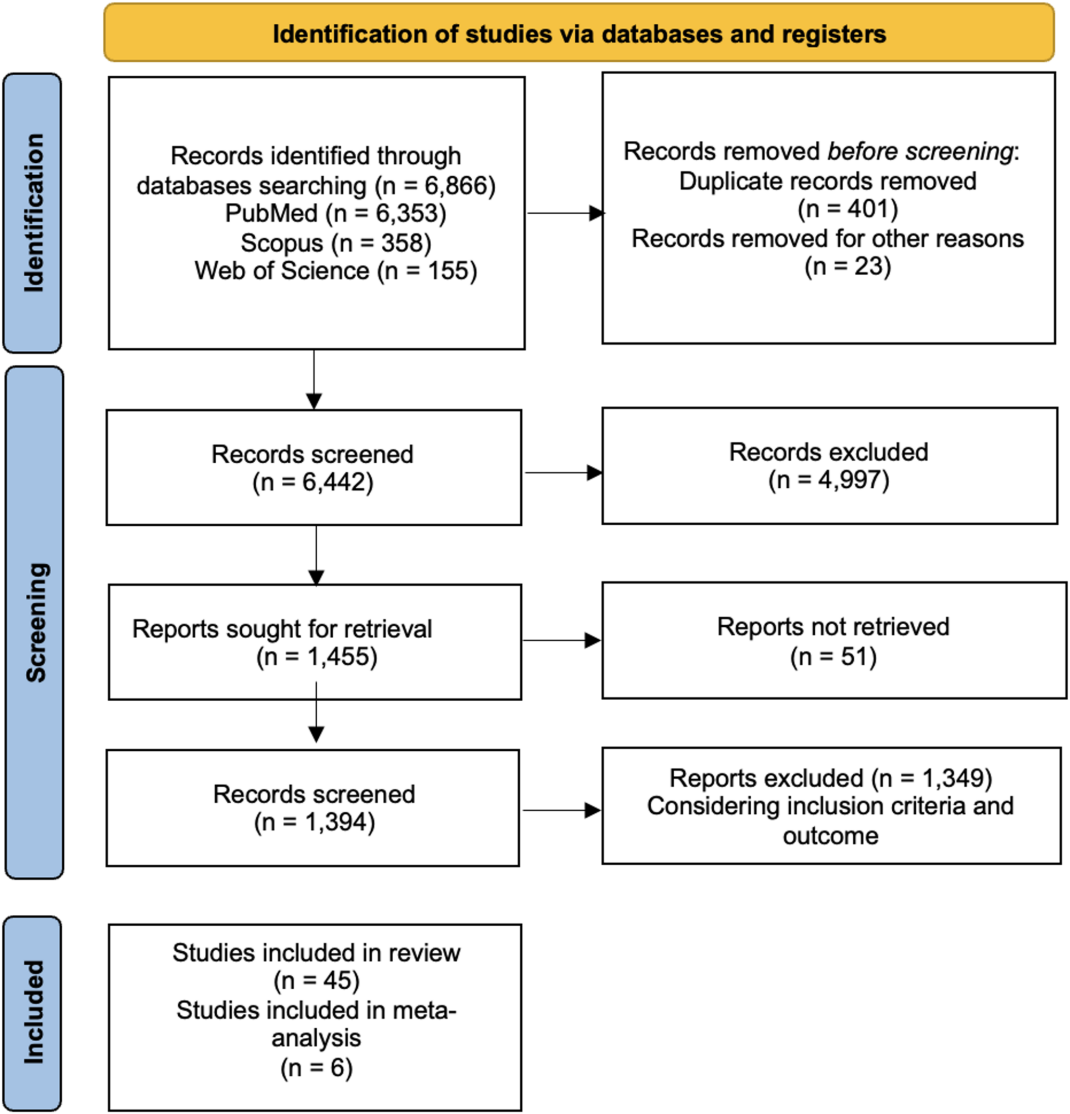
Flowchart of the research of eligible studies on the impact of thyroid function on psychiatric diseases

A total of 6,866 articles were identified through the computer literature search. By reviewing the titles and abstracts, 4,997 articles were excluded because the reported data were not within the field of interest of this review. Consequently, 45 articles were selected, retrieved in full-text versions and evaluated in the review (Fig. 1).

**Fig. 2:**
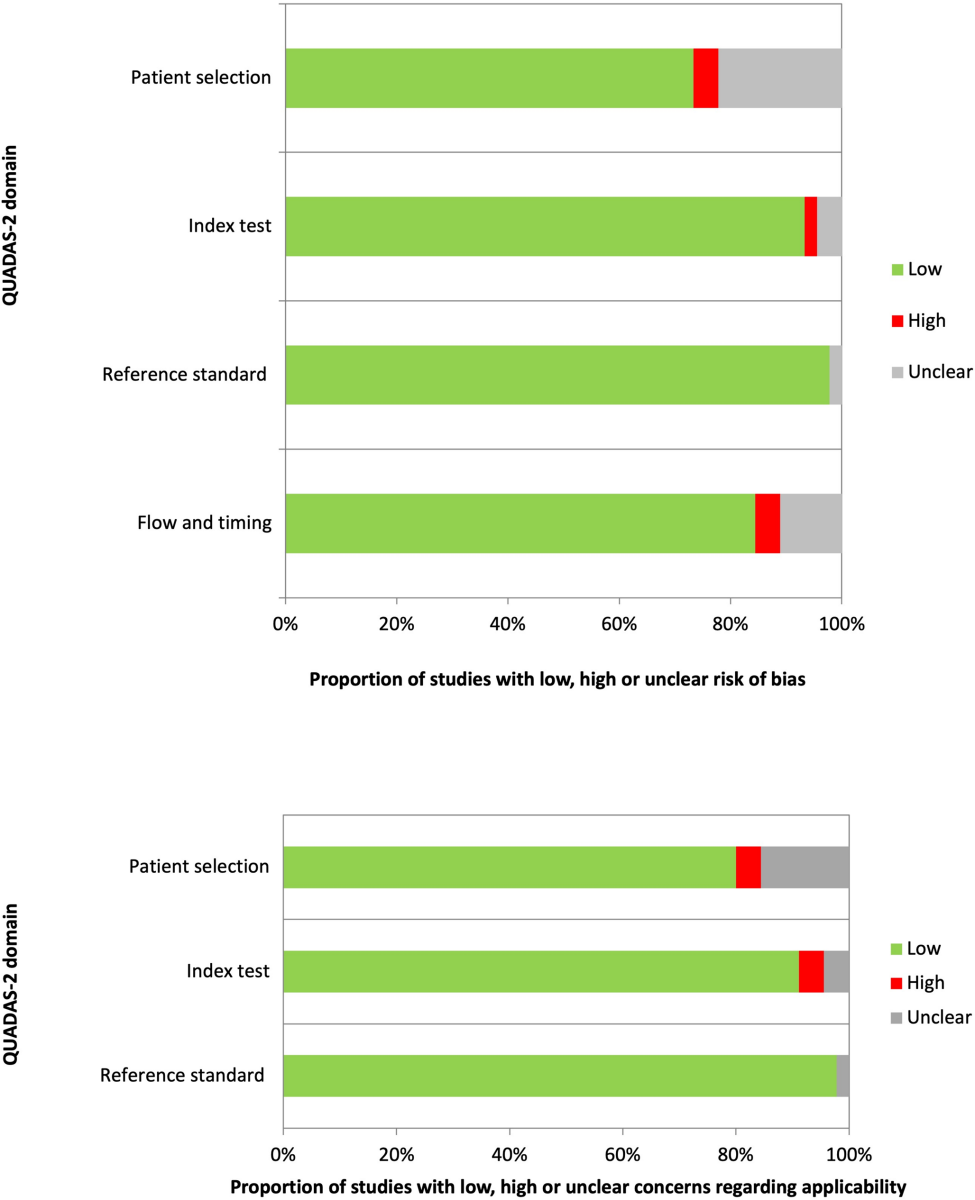
QUADAS-2 quality assessment for risk of bias and applicability concerns for the studies considered in the review

In general, the quality assessment using QUADAS-2 evaluation underlined the presence of unclear risk of bias and applicability concerns in some of the studies for what concerns patients’ selection, index test, reference standard and flow and timing. Nevertheless, only a small number of studies were characterized by the presence of high risks of bias or applicability (Fig. 2).

The main characteristics of the studies and their results are briefly presented in Tables 1 and 2.

**Table 1:** Characteristics of the human studies considered for the review

First author	Ref. *N*.	Year	Country	Study design	*N*. Pts.	Age	Sex M: F
Banki	[32]	1984	Hungary	Interventional	17	41 ± 13	0/17
Kjellman	[25]	1984	Sweden	Prospective cross-sectional	32	43 ± 1.9	18/14
Undèn	[58]	1987	Sweden	Interventional	26	43.5 ± 1.9	11/15
Wahby	[65]	1988	United States	Interventional	41	45.3 ± 2.0	41/0
Wahby	[31]	1989	United States	Prospective cross-sectional	27	36.9 ± 2.9	27/0
Poirier	[30]	1994	France	Prospective cross-sectional	55	47 ± 12	20/35
Medici	[66]	2014	The Netherlands	Prospective observational	1503	70.6 ± 7.3	576/927
Wysokiński	[67]	2014	Poland	Prospective cross-sectional	652	52.1 ± 21.7	158/494
Degner	[34]	2015	Germany	Prospective cross-sectional	19	40.5	18/1
Asadikaram	[28]	2019	Iran	Retrospective cross-sectional	79	36.1 ± 16.6	40/39
Mokrani	[37]	2020	France	Interventional	145	35.9 ± 10.0	70/75
Makarow-Gronert	[68]	2021	Poland	Retrospective cross-sectional	179	NA	31/148
Zhou	[27]	2021	China	Retrospective cross-sectional	537	36.15 ± 13.59	201/336
Liu	[35]	2022	China	Retrospective cross-sectional	723	63.3 ± 8.7	279/444
Ma	[44]	2022	China	Retrospective cross-sectional	80	15.3 ± 2.0	80/0
Shangguan	[39]	2022	China	Prospective cross-sectional	1936	34.8 ± 12.4	647/1289
Zhao	[69]	2022	China	Prospective cross-sectional	30	50.85 ± 4.69	10/20
Zhu	[70]	2022	China	Retrospective cross-sectional	1137	32.68 ± 11.29	506/631
Dai	[38]	2023	China	Retrospective observational	481	19.6 ± 3.6	196/285
Feng	[71]	2023	China	Prospective cross-sectional	1718	34.79 ± 12.36	588/1130
Hu	[72]	2023	China	Retrospective cross-sectional	1251	28.81 ± 8.36	458/793
Huang	[73]	2023	China	Prospective cross-sectional	1369	39 (29–48)	439/930
Jiang	[43]	2023	China	Retrospective cross sectional	765	28.9 ± 8.6	370/595
Li	[42]	2023	China	Prospective cross-sectional	116	24.3 ± 5.1	36/80
Liang	[36]	2023	China	Prospective cross sectional	67	39.0 (IQR 31.5–52.0)	20/47
Peng	[74]	2023	China	Prospective cross-sectional	1706	34 (IQR 23–45)	1120/586
Peng	[75]	2023	China	Retrospective observational	1718	34 (IQR 23–45)	588/1130
Peng	[76]	2023	China	Retrospective observational	1718	34 (IQR 23–45)	588/1130
Peng	[77]	2023	China	Prospective cross-sectional	1130	35.8 ± 12.4	0/1130
Qi	[78]	2023	China	Prospective cross-sectional	981	34 (IQR 18–62)	333/648
Song	[79]	2023	China	Retrospective cross-sectional	1076	NA	374/702
Sun	[29]	2023	China	Prospective cross-sectional	894	35.3 ± 7.5	295/599
Tang	[80]	2023	China	Prospective cross-sectional	981	35.62 ± 12.44	333/648
Yang	[81]	2023	China	Prospective cross-sectional	679	14.55 ± 1.59	139/540
Yang	[41]	2023	China	Retrospective observational	1718	38.87 ± 12.43	588/1130
Yang	[82]	2023	China	Retrospective cross-sectional	1718	34.66 ± 12.43	NA
Ye	[45]	2023	China	Retrospective observational	467	25.6 ± 5.5	164/303
Zhan	[83]	2023	China	Prospective observational	708	36.09 ± 1.60	230/479
Zhang	[84]	2023	China	Retrospective observational	179	15.40 ± 1.56	51/128
Zhao	[59]	2023	China	Prospective cross-sectional	1289	29.3 ± 8.6	470/819
Zheng	[85]	2023	China	Retrospective observational	1718	34.9 ± 12.3	588/1130
Cui	[33]	2024	China	Prospective cross-sectional	1236	32.40 ± 11.44	507/729
Luo	[40]	2024	China	Prospective cross-sectional	1380	34.96 ± 12.48	475/905
Kumaratne	[86]	2024	United States	Prospective cross-sectional	125	15.5 ± 2.0	44/81
Yang	[26]	2024	China	Prospective cross-sectional	42	26.43 ± 10.79	15/27

Ref.: references; N.: number; Pts.: patients; M: male; F: female; NA: not available

**Table 2:** Results and main findings of the human studies considered for the review

First author	Patients’ characteristics	TSH levels	Main findings
Asadikaram [28]	Adult outpatients diagnosed with MDD	1.4 ± 1.5	TSH levels were significantly lower in MDD patients compared to healthy controls
Banki [32]	Hospitalized drug-naïve adult women	1.89 ± 1.37	No differences in TSH levels were found among MDD and healthy controls
Cui [33]	Drug-free aged below 50 adult patients	1.93 ± 1.59	No differences in TSH levels were found among MDD and healthy controls
Dai [38]	Drug-naïve young adult outpatients diagnosed with MDD	4.82 ± 2.07	Among MDD patients with SCH, those without symptoms had significantly lower serum TSH levels
Degner [34]	Adult outpatients without previously diagnosed thyroidal diseases.	1.6 ± 1.5	No differences in TSH levels were found among BD, MDD and SHZ patients; AbTPO levels were higher in MDD and BD compared to SHZ
Feng [71]	Adult outpatients diagnosed with MDD.	6.30 ± 2.79	MDD patients with suicide attempt had elevated serum TSH levels
Hu [72]	Adult Chinese first-episode, drug-naïve outpatients	4.93 ± 2.39	TSH levels are implicated in abnormal lipid metabolism in young patients with first-episode, drug-naïve MDD
Huang [73]	Adult inpatients diagnosed with MDD.	SA 6.7 (4.39–8.86) NSA 4.73 (2.9–6.22)	MDD patients with suicide attempt had elevated serum TSH levels
Jiang [43]	Adult Chinese first-episode, drug-naïve MDD patients	5.6 ± 2.4	MDD patients with suicide attempt had elevated serum TSH levels
Kjellman [25]	Adult patients admitted for acute MDD	2.6 ± 0.1	Significantly lower serum levels of TSH and less variability of TSH levels during the 24 h period were found in acute MDD patients compared to controls
Kumaratne [86]	Adolescent patients diagnosed with MDD	1.91 ± 1.02	There is no statistically significant association between depression and subclinical hypothyroidism
Liang [36]	Adult Chinese first-episode, drug-naïve MDD patients aged 18 to 65	1.59 (IQR 0.98–2.27)	No differences in TSH levels were found among MDD and healthy controls
Li [42]	Adult Chinese first-episode, drug-naïve MDD patients	7.0 ± 2.6	Young MDD with high rate of SA had lower TSH serum levels
Liu [35]	Adult Chinese multi-ethnic MDD outpatients	3.4 ± 3.3	No differences in TSH levels were found among MDD patients and healthy controls
Luo [40]	Adult chinese first-episode, drug-naïve MDD patients	5.29 ± 2.69	MDD patients with suicide attempt had elevated serum TSH levels
Ma [44]	Adolescent Chinese first-episode, drug-naïve MDD inpatients	3.23 ± 1.27	MDD patients with suicide attempt had elevated serum TSH levels
Makarow-Gronert [68]	Caucasian patients aged 12 to 18 years who were hospitalized in the Department of Adolescent Psychiatry	2.08 ± 1.02	There may be a higher prevalence of thyroid dysfunctions in BP and MDD subgroups among Adolescents
Medici [66]	Caucasian aged 55 years older from Rotterdam	1.30 (0.90-2.00)	Elderly persons with low-normal TSH a substantially increased risk of developing a depressive syndrome in the subsequent years
Mokrani [37]	Adult MDD drug-free for at least two weeks who underwent TRH-TSH stimulation	1.04 ± 1.54	MDD patients showed reduced TRH-TSH response compared to healthy controls
Peng [74]	Adult Chinese first-episode, drug-naïve outpatients aged 18 to 60	4.91 (3.11, 6.66)	SCH might play a vital role in the relationship between metabolic syndrome and MDD symptoms
Peng [75]	Adult Chinese first-episode, drug-naïve patients aged 18 to 60	4.91 (IQR 3.11–6.66)	MDD patients may have thyroid dysfunction in the early stage. SCH may hold promise in reducing clinical symptoms
Peng [76]	Adult Chinese first-episode, drug-naïve outpatients aged 18 to 60	4.91 (IQR 3.11–6.66)	MDD patients with suicide attempt had significantly higher TSH levels
Peng [77]	Adult Chinese first-episode, drug-naïve female patients	5.13 ± 2.45	Higher TSH levels were correlated with psychotic symptoms in female MDD patients
Poirier [30]	Adult MDD inpatients	0.98 ± 1.1	Serum TSH levels were significantly lower in the MDD compared with healthy controls
Qi [78]	First-time inpatients with MDD.	3.98 ± 2.47	TSH levels are a risk factor for hypoglycemia in MDD patients
Shangguan [39]	Drug-naïve MDD patients aged 16 to 60 years.	SA 6.8 (0.4–13) NSA 4.6 (0.4–12.6)	SCH comorbidity may pose a specific hazard in MDD patients due to increased suicide attempts
Song [79]	Drug-naïve MDD outpatients aged 18 to 45.	SA 6.6 (IQR 3.87–8.55) NSA 3.93 (2.61–5.65)	MDD patients with suicide attempt had elevated serum TSH levels
Sun [29]	Adult Chinese first-episode, drug-naïve patients aged 18 to 60	5.60 ± 2.82	Early-adult onset MDD patients had lower TSH levels compared to mild-adult onset MDD patients
Tang [80]	MDD patients	3.48 ± 2.47	Higher TSH levels were risk factors for the development and the severity of metabolic syndrome in MDD patients
Undèn [58]	Drug-free hospitalized MDD patients	2.9 ± 0.2	MDD patients showed lower TSH levels, due to a downregulation of the pituitary TRH receptors
Wahby [65]	Drug-free washout adult male patients who underwent TRH-TSH stimulation	3.6 ± 0.3	Schizodepressed patients appeared significantly different from MDD but closer to SHZ and healthy controls on the TRH test
Wahby [31]	Adult MDD patients	3.0 ± 0.2	MDD patients showed significantly higher serum TSH levels
Wysokiński [67]	Hospitalized patients in acute phase evaluated at first entry	1.63 ± 1.95	Compared with BD, patients with MDD have the lowest level of TSH
Yang [82]	Adult Chinese first-episode, drug-naïve patients	5.069 ± 2.561	Comorbid anxiety MDD patients is positively associated with elevated TSH; clinical treatment of impaired thyroid function may be useful for comorbid anxiety
Yang [81]	Adolescent inpatients and outpatients aged 12 to 18 years	NA	Patients with psychotic symptoms showed decreased fT4
Yang [41]	Adult Chinese first-episode, drug-naïve outpatients	5.07 ± 2.56	MDD patients with suicide attempt had significantly higher TSH levels
Yang [26]	Adult MDD patients aged 18 to 60 years	1.65 ± 0.83	MDD patients had significantly lower TSH and fT3 levels
Ye [45]	Adult Chinese first-episode, drug-naïve outpatients	5.34 ± 2.68	MDD patients with suicide attempt had significantly higher TSH levels
Zhan [83]	Adult Chinese first-episode, drug-naïve female inpatients	1.19 ± 2.64	Higher TSH levels were correlated with psychotic symptoms in female MDD patients
Zhang [84]	Adolescents with MDD from outpatients and inpatient	1.25 ± 0.24	TSH levels were significantly higher in Suicide attempters
Zhao [69]	Middle age drug naïve MDD patients	1.56 ± 0.58	There are changes in prefrontal cortex and cerebellar biochemical metabolism in patients with MDD, which may be related to cognitive disorder or altered TSH levels
Zhao [59]	Adult outpatients with MDD	4.9 ± 2.5	MDD patents experiencing anxiety showed higher TSH levels
Zheng [85]	Adult outpatients with MDD	5.07 ± 2.41	Higher TSH levels were risk factors for the development of metabolic syndrome in MDD patients
Zhou [27]	MDD inpatients aged 16 to 65 years	1.52 (IQR 1.09–2.36)	TSH, fT4 and fT3 levels were significantly lower in MDD patients when compared to controls
Zhu [70]	Hospitalized mood disorder patients	2.38 ± 2.90	FT4, and FT3 secretion differed between BD and MDD, whereas TSH secretion differed only in the male subgroup

TSH: thyrotropin stimulating hormone; fT4: free thyroxine; fT3: free triiodothyronine; BD: bipolar disorder; MDD: major depressive disorder; SCH: subclinical hypothyroidism; TRH: thyrotropin-releasing hormone; SA: suicide attempt; NSA: non suicide attempt

### Qualitative analysis

The available data on 34,448 (34% male) patients affected by MDD were retrieved from 45 studies: 17 retrospectives in nature, 24 prospective and four interventional. The studies were conducted in different Countries: Thirty-three from Asia, nine from Europe, and three from North America. The data are summarized in Tables 1 and 2.

In the 1980 s, Kjellman et al. showed that adult hospitalized MDD patients had less variability in TSH serum levels over a 24-hour period compared to healthy controls. In the same study, the authors found significantly lower serum TSH levels in MDD patients compared to controls (2.6 ± 0.1 mIU/L vs. 3.6 ± 0.2 mIU/L, respectively, *p* <.001) [25]. These data were confirmed in many subsequent reports [26–30]. Only Wahby et al. showed in a small set of male patients higher TSH levels in MDD compared to healthy controls (3.0 ± 0.2 mIU/L vs. 2.5 ± 0.6 mIU/L, respectively, *p* <.005) [31]. A few other studies did not show any significant difference in serum TSH levels between drug-naïve MDD patients and healthy controls [32–36]. Mokrani et al. showed that MDD patients had a reduce TRH-TSH response compared to healthy subjects (ΔTSH 1.99 ± 1.62 mIU/L vs. 3.75 ± 1.40 mIU/L, respectively, p = < 0.001) [37]. Going on, Recently, Dai et al. showed that drug-naïve MDD patients with higher TSH levels may suffer from more psychotic symptoms (7.57 ± 3.23 mIU/L vs. 5.99 ± 2.45 mIU/L, p = < 0.001) [38]; in the same vein, Shangguan et al., in a large series of patients (1706 subjects, 586 male), stated that those with suicide attempts (SA) had higher TSH levels than those without [median: 6.8 mIU/L (IQR 0.4–13) vs. 4.6 mIU/L (IQR 0.4–12.6), respectively, p = < 0.001] [39]. Luo et al. confirmed these data in 1380 (475 male) drug-naïve adult Chinese MDD subjects (6.78 ± 2.91 mIU/L vs. 4.8 ± 2.4 mIU/L, respectively, *p* <.001) [40].

### Quantitative analysis

**Fig. 3:**
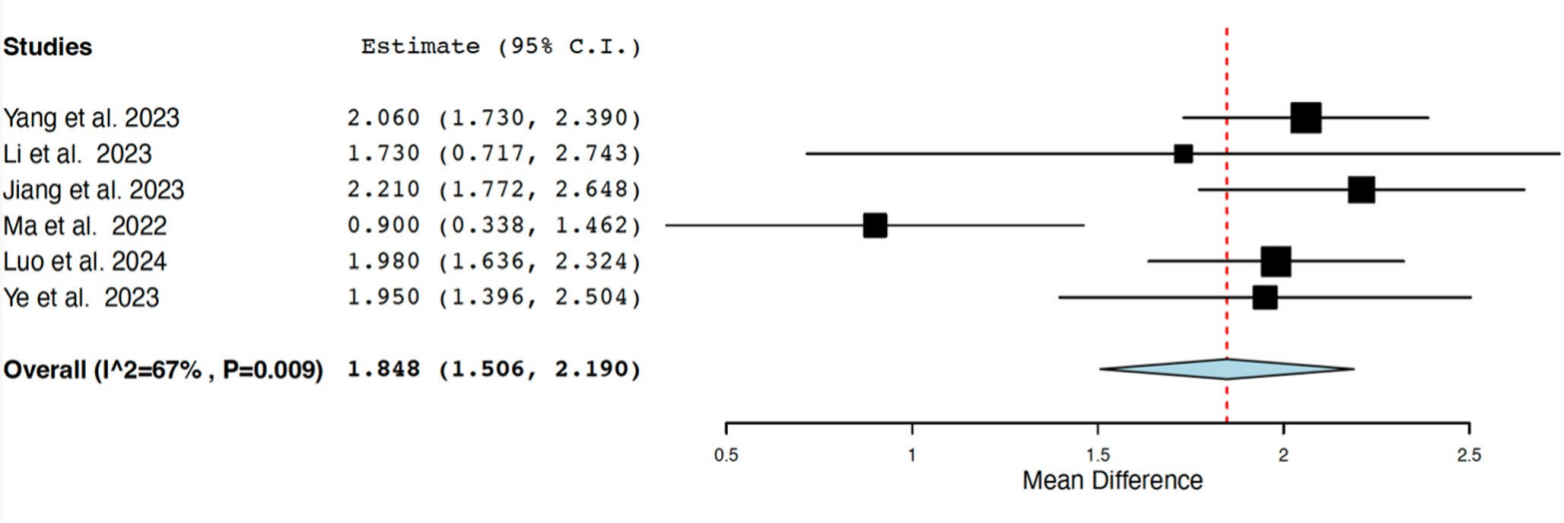
Forest plot of the meta-analysis on serum concentrations of thyroid stimulating hormone (TSH) in patients with major depressive disorder with suicide attempt compared with patients with major depressive disorder without suicide attempt

To evaluate any TSH differences among MDD patients with or without SA, six studies [40–45] were pooled for a total of 6,224 patients (2,447 with SA and 3,797 without SA). All studies were from Asia, three were retrospective and three prospective. The meta-analysis showed that SA patients had higher serum TSH levels compared to those without SA (Standardized Mean Difference = 1.848 mIU/L, C.I. 95%:1.506 to 2.190) with moderate-high heterogeneity across studies (I^2^ = 67%, *p* =.009) (Fig. 3).

### Animal studies

A total of 9 studies evaluating thyroid disfunction related to depression and anxiety-like behavior were found and summarized in Table 3.

**Table 3:** Results and main findings of the animal studies considered for the review

First author	Ref. *N*.	Year	Animal model	Main findings
Lifschytz	[50]	2004	Male albino rats (Sabra strain, derived from Wistar strain)	The combination of fluoxetine and T3 induced desensitization of 5-HT_1B_ autoreceptors in hypothalamus, augmenting and accelerating the effect of the SSRI
Venero	[13]	2005	TRα1 mutated mice	T3 treatment ameliorated anxiety, memory impairment, and locomotor dysfunction hypothyroidism related
Pilhatsch	[51]	2010	TRα1 mutated mice	Continuous T3 infusion reverse depressive and anxious behavior. Iatrogeneously induced hyperthyroidism by continuous T3-administration leads to a hypolocomotive and depressive phenotype
Siqueira	[87]	2010	Male Wistar stars treated with MTZ	Selective attenuation by MTZ of DPAG-evoked defensive behaviors supports attenuation of panic attacks in hypothyroidism
Ge	[48]	2013	Adult Wistar rats with clinical and subclinical hypothyroidism	SCH could result in depression-like behavior, accompanied by subtle hyperactivity of HPA axis
Yu	[49]	2015	Male Sprague-Dawley rats treated with ^131^iodine or L-T4	Hypothyroid rats exhibited decreased depression- and anxiety-like behavior; hyperthyroid rats showed higher ones
Bárez-López	[47]	2017	Knockout mice lacking D2 (D2KO)	Hippocampal hypothyroidism downregulates the GABAergic transmission resulting in anxiety
Russart	[52]	2018	Epac1^−/−^, Prkar1a^−/−^ and R1A-Epac1KO mice	Hyperthyroidism is related to increased aggressive behavior
Maglione	[14]	2022	Male transgenic 3xTg-AD (APPswe, PS1m146v, tauP301L)	In an Alzheimer’s disease mouse model, T3 supplementation promotes improvements in depression-like behavior, and attenuated disease progression

MTZ = methimazole; D2 = deiodinases 2; DPAG = dorsal periaqueductal gray matter; 5-HT = serotonin; 5-HT_1B_ = 5-hydroxytryptamine receptor 1B; 5-HT_1A_ = 5-hydroxytryptamine receptor 1 A; SSRI = serotonin re-uptake inhibitor; T3 = triiodothyronine; TPOAb = thyroid peroxidase antibodies; L-T4 = levothyroxine; SCH = subclinical hypothyroidism; HPA = hypothalamus-pituitary-adrenal

Baréz-Lopéz et al. showed increase anxiety in knockout mice lacking deiodinase 2 (D2KO), which are characterized by high levels of TSH and 50% reduction of the brain T3 content [46, 47]. The authors hypothesized that the hypothyroid status in the hippocampus and amygdala can alter GABAergic transmission and/or cause desynchronized activity between the brain regions involved in anxiety [47]. In addition, Ge et al. evidenced that subclinical hypothyroid mice, obtained by hemi-thyroid electrocauterization, exhibit depression-like behavior due to low T3 levels in hippocampus [48]. On the contrary, Yu et al. evidenced in hypothyroid Male Sprague–Dawley rats induced by 131-Iodine decreased anxiety and depression-like behaviors [49]. Anyway, all studies on T3 administration showed improvement in these parameters [13, 14, 50, 51]. Twenty years ago, Lifschtz et al. demonstrated that the administration T3 enhances the beneficial effect of fluoxetine by desensitizing the 5-hydroxytryptamine receptor 1B in hypothalamus [50]. Pilhatsch et al. studied the effects of continuous T3 infusion in mutant thyroid hormone receptor alpha 1 (TRα1) mice, which are characterized by receptor-mediated hypothyroidism in the brain leading to depressive and anxious behavior. They showed a significant improvement in these disturbances with T3 infusion [51]. Maglione et al. showed in a male Alzheimer’s disease (AD) mouse model that T3 therapy improves the depression-like behavior observed in AD transgenic mice. This effect is mediated by the modulation of serotonergic-related genes involved in transmission via serotonin receptors 1 A (5HT1A) and serotonin reuptake [14]. Venero et al. found that anxiety and memory deficiencies were relieved by treatment with high levels of T3 in adult TRα1 mice, correlated with the normalization of GABAergic inhibitory interneurons in the hippocampal CA1 region [13].

Only one study explored the relationship between hyperthyroidism with suppressive TSH levels and emotional distress. Russart et al. showed that hyperthyroidism is related to increased aggressive behavior in male adult mice, hypothesizing a cross talk between altered thyroid signaling and aggressive behavior [52].

## Discussion

Based on our analysis of the literature, an interplay between MDD and TSH levels seems to emerge, corroborating animal models. In addition, our meta-analysis showed that higher TSH levels are related to an increased risk of SA among MDD patients.

The bridge that arrows thyroid function and the brain is well known. Even in the early stages of embryogenesis, thyroid hormones (TH) are crucial for the development of the mammalian brain, influencing the migration and differentiation of neural cells, synaptogenesis, and myelination [53]. Moreover, thyroid function is related to short- and long-term brain changes including neuronal plasticity processes, angiogenesis and neurogenesis in adults [54]. It is well recognized that TH play a modulatory role in affective disorders by enhancing serotonergic neurotransmission. This occurs through a reduction in the sensitivity of the 5-HT1A receptor in the raphe region and an increase in the sensitivity of the 5-HT2 receptor [16]. Furthermore, TH receptors are found in limbic structures involved in mood regulation [55], and thyronine has the ability to specifically interact with various neurotransmitter receptors, including those associated with GABAergic, catecholaminergic, glutamatergic, and cholinergic systems [17]. Hence, it has been hypothesized that TH could be considered neurotransmitters, although they do not satisfy all the criteria for this definition at the current state of research [17]. Nevertheless, it is clear that TH play a role in regulating behavior and mental state, including mood modulation. Overt hypothyroidism frequently manifests as cognitive impairment, distress, and sadness, whereas hyperthyroidism can result in agitation, severe psychosis, and apathy, particularly in the elderly [4]. Different levels of serum TSH are associated with varying prevalence of comorbidities [56, 57], and it is reasonable to think that this is appliable also to psychiatric disorders. It is quite common to be affected by both thyroid dysfunction and psychiatric disorders concomitantly, and the former could influence or imbalance the latter.

Animal studies obtained in knockout mice lacking D2KO suggested that increased TSH levels, due to reduced T3 in the brain, are associated with increased anxiety [46, 47]. The same result was observed in subclinical hypothyroid mice obtained by hemi-thyroid electrocauterization [48]. Interestingly, all available data in animals clearly showed that T3 administration ameliorated anxiety and depressive behavior [13, 14, 51] and improved the beneficial effects of antidepressants [50]. This effect seems to be mediated by the modulation of serotonin uptake and/or GABAergic activity in the hippocampal region [13, 14].

Our systematic review showed conflicting data about TSH levels in FEDN MDD patients compared to healthy subjects: some studies exhibit no differences [32, 33, 36], whereas many others showed lower TSH levels [25–28, 30, 38, 58]. At this moment, the question of whether TSH levels differ between FEDN patients diagnosed with MDD and healthy subjects remains unresolved.

However, answering this question could be challenging because patients with episodes of MDD may also have concomitant non-thyroidal illness syndrome.

Going on, all recent literature data underline that, among MDD patients, those exhibiting a significantly higher TSH levels suffer from more psychotic symptoms, severe depressive behavior and more important a higher rate of suicide attempt [38–40, 59]. This last issue was completely corroborated by our meta-analysis. In fact, we showed that MDD patients with SA exhibited higher TSH level than those without SA (Fig. 3). Again, we must underline that also these data could be affected by the above non-thyroidal illness syndrome. However, the TSH levels were obtained in patients all at disease onset, and there is no reason to believe that the group of patients with suicide attempts, or vice versa, has a higher prevalence of non-thyroidal illness syndrome.

Nevertheless, the clinical implications of this finding have yet to be established. Specifically, can L-T4 or thioamide treatment modulate the behavioral manifestations of MDD patients? What are the optimal TSH levels for these patients? Should we now consider tailoring TSH targets based on individual MDD phenotypes? At the moment we can only show that LT4 treatment could improve depressive symptoms, therapeutic response to tricyclic anti-depressant [60–64].

It is important to underline that a limitation of this review is the lack of a pre-registered protocol. Although the methodology was defined a priori by all authors, the absence of a publicly accessible protocol may limit the transparency and reproducibility of the review process.

In conclusion, this systematic review showed conflicting data about TSH levels in FEDN MDD patients compared to healthy subjects, but the meta-analysis evidenced significant higher TSH levels among MDD patients with suicide attempt than those without it. The clinical implications of this finding have yet to be established.

## Data Availability

Original data analysed during this study are included in this published article.
